# P-2003. Clinical Outcomes of COVID-19 among Immunocompromised Patients at Siriraj Hospital

**DOI:** 10.1093/ofid/ofae631.2160

**Published:** 2025-01-29

**Authors:** Pakpoom Phoompoung, Jirapa Dilokruangchai

**Affiliations:** Siriraj Hospital / Mahidol University, Thailand, Bangkok, Krung Thep, Thailand; Faculty of Medicine Siriraj Hospital, Mahidol University, Bangkoknoi, Krung Thep, Thailand

## Abstract

**Background:**

The coronavirus disease 2019 (COVID-19) pandemic has caused significant mortality for patients worldwide. The clinical outcomes of COVID-19 among immunosuppressed patients, who are at presumably risk of more severe disease have not been well characterized.

Clinical outcome between immunocompetent and immunocompromised patients hospitalized with COVID-19

**Figure:**
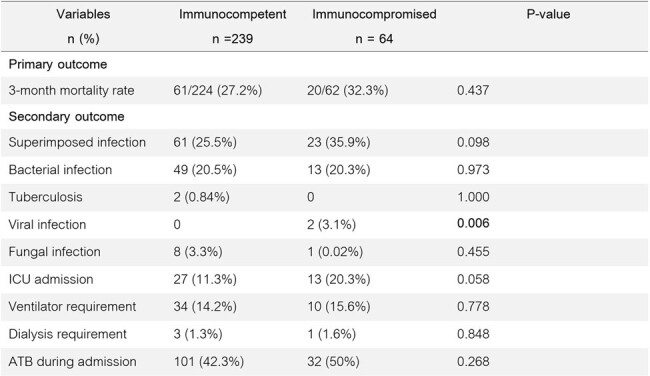

**Methods:**

We performed a retrospective cohort study including all adult patients hospitalized with COVID-19 during July to December 2021. Primary outcome was a 3-month death among immunocompetent and immunocompromised patients. Secondary outcomes included superimposed infections and intensive care unit (ICU) admission within a 6-month period, and factors associated with death in immunocompromised patients.

Comparison between immunocompromised patients hospitalized with COVID-19 who were dead and survived during 3 months after COVID-19

**Figure:**
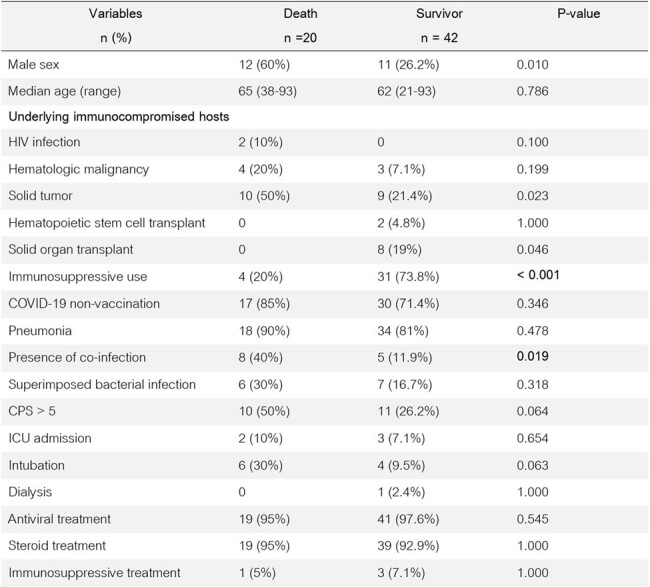

**Results:**

303 patients were enrolled (239 immunocompetent and 64 immunocompromised patients, with mean age of 71 (±14) and 63 (±16) years, respectively). Immunocompromised patients showed more superimposed herpes simplex virus infection (3.1% vs. 0%, P 0.006). There was a trend towards higher ICU admission rate (20.3% vs. 11.%, P 0.058) in the immunocompromised group. However, the 3-month death between both groups did not differ significantly (24.7% immunocompromised vs 20.5% immunocompetent, P 0.437). Interestingly, there was a higher death among immunocompromised patients who had co-infections (61.5% vs. 32.4%, P 0.019) and superimposed infections (56.5% vs. 17.9%, P 0.002). On the other hand, there was a lower death rate in immunocompromised patients receiving immunosuppressive drugs prior to COVID-19 diagnosis (12.9% vs. 59.2%, P < 0.001).

**Conclusion:**

Three-month mortality rate did not differ between immunocompromised and immunocompetent patients. Superimposed and co-infections increased mortality in immunocompromised COVID-19 patients. Prior use of immunosuppressive agents was associated with less mortality, which may be explained by less detrimental inflammatory response.

**Disclosures:**

All Authors: No reported disclosures

